# Modulatory Effects of Tea Components with Different Fermentation Degrees on Fluoride Bioavailability in Rats

**DOI:** 10.3390/foods15060984

**Published:** 2026-03-10

**Authors:** Jingjing Li, Zhichao Xu, Yanan Hu, Ying Huang, Pengcheng Hu, Chaoyuan Hou, Ruyan Hou, Chuanyi Peng, Daxiang Li, Xiaochun Wan, Guijie Chen, Huimei Cai

**Affiliations:** 1 State Key Laboratory for Tea Plant Germplasm Innovation and Resourse Utilization, Anhui Agricultural University, Hefei 230036, China; 5001510@ahau.edu.cn (J.L.); 15955169503@163.com (Z.X.); hywyzh2@163.com (Y.H.); 13399511143@163.com (P.H.); hcy904520@gmail.com (C.H.); hry@ahau.edu.cn (R.H.); pcy0917@ahau.edu.cn (C.P.); dxli@ahau.edu.cn (D.L.); xcwan@ahau.edu.cn (X.W.); 2 Anhui Provincial Key Laboratory of Food Safety Monitoring and Quality Control, Joint Research Center for Food Nutrition and Health of IHM, School of Food and Nutrition, Anhui Agricultural University, Hefei 230036, China; 3 School of Tea Science, Anhui Agricultural University, Hefei 230036, China; 4 College of Food Engineering, Ningxia Minjiang University of Applied Technology, Shizuishan 753000, China; 18409573505@163.com

**Keywords:** fluoride bioavailability, pharmacokinetics, epigallocatechin gallate (EGCG), theapigments, tea polysaccharides, mineral elements, *Camellia sinensis*

## Abstract

Tea offers health benefits, but some teas accumulate high fluoride (F), posing fluorosis risks. However, the roles of individual tea components in regulating F bioavailability remain unclear. This study investigated the effects of major tea constituents on F metabolism in male rats (*n* = 5/group) administered F (40 mg/L) alone or with graded doses of epigallocatechin gallate (EGCG, 150–450 mg/kg); theaflavins, thearubigins, and theabrownin (TFs, TRs, TB, 200–800 mg/kg each); tea polysaccharides (TPSs, 25–250 mg/kg); and calcium and aluminum (Ca, Al, 800–3200 µg/kg each) via gavage. Pharmacokinetic analysis of plasma F (0–480 min) and fecal F excretion were assessed. The result showed that high-dose EGCG (450 mg/kg) reduced C_max_ by 61.76% and total exposure (AUC_0__–t_) by 37.48% compared to the control, while significantly increasing fecal F by 26.79% (*p* < 0.05). TB (800 mg/kg) delayed F absorption by prolonging T_max_ from 18 to 30 min and reduced C_max_ by 35.38% (*p* < 0.05). TPS (250 mg/kg) decreased C_max_ by 51.72% and AUC_0–t_ by 24.38% (*p* < 0.05). Ca and Al (800–3200 µg/kg) reduced C_max_ by 39.19–69.62%, and low-dose aluminum (800 µg/kg) increased fecal F by 35.58% (*p* < 0.05). These findings elucidate distinct roles of tea constituents in mitigating F bioavailability, providing a scientific basis for tea safety assessment and dietary interventions against F overexposure.

## 1. Introduction

Tea is one of the most widely consumed beverages worldwide, and its health benefits, including antioxidant [1], anti-aging [2], and antitumor effects [3], are well documented. These beneficial properties are largely attributed to the diverse functional components present in tea leaves [4,5], such as tea polyphenols (e.g., epigallocatechin gallate, EGCG), amino acids, tea polysaccharides (TPSs), theapigments (thearubigins, TRs; theaflavins, TFs; theabrownin, TB), and various mineral elements. However, tea plants (*Camellia sinensis*) are known to accumulate fluoride (F) from soil [6], with mature leaves and certain fermented teas, such dark tea and brick tea, containing particularly high levels [7]. While moderate F intake helps prevent dental caries [8], excessive long-term consumption can lead to dental and skeletal fluorosis [9,10]. Importantly, the health impact of F from tea consumption depends not only on total F ingested but also on its bioavailability—the fraction absorbed and physiologically available [11].

Tea components may interact with F in the gastrointestinal tract through various mechanisms. For instance, polyphenols can bind F via hydrogen bonding [12], while calcium (Ca) and aluminum (Al) form insoluble F salts under physiological pH [13,14]. These interactions could potentially modulate F absorption and excretion. Previous studies have investigated F interactions with specific dietary components or the effects of whole tea matrices [13,14]. However, a systematic, component-level comparison of how major tea constituents independently influence F pharmacokinetics has little been reported. Furthermore, no study has applied pharmacokinetic modeling to compare the effects of these components on F absorption, distribution, and excretion under identical experimental conditions.

The rat model is well-established for F pharmacokinetic studies, with comparable gastrointestinal physiology, F absorption kinetics (primarily passive diffusion), and renal handling to humans [15], providing a reliable basis for extrapolating findings to human health risk assessment. We hypothesized that different tea components would exert distinct, dose-dependent effects on F absorption and excretion, with some components (e.g., Ca, Al) reducing bioavailability through precipitation, while others (e.g., TPs, TB) might modulate F kinetics via complexation or delayed absorption. Therefore, this study aimed to systematically evaluate the regulatory effects of key tea components, including EGCG, TFs, TRs, TB, TPS, Ca, and Al, on F absorption and metabolism using a rat pharmacokinetic model. These findings will contribute to a deeper understanding of how different tea types may differentially influence F bioavailability and provide essential data for accurate F risk assessment.

## 2. Materials and Methods

### 2.1. Chemicals and Reagents

Sodium fluoride (NaF), sodium hydroxide (NaOH), trisodium citrate dihydrate (Na_3_C_6_H_5_O_7_·2H_2_O), sodium chloride (NaCl), acetic acid (CH_3_COOH), hydrochloric acid (HCl), calcium chloride (CaCl_2_), and aluminum chloride (AlCl_3_) were purchased from Sinopharm Chemical Reagent Co., Ltd. (Shanghai, China). Chloral hydrate (C_2_H_3_Cl_3_O_2_) was obtained from Soleibao Technology Co., Ltd. (Beijing, China). EGCG, TFs, TRs, and TB were supplied by Shengyuan Biotechnology Co., Ltd. (Weifang, China). TPSs were purchased from Yuanye Biotechnology Co., Ltd. (Shanghai, China). All reagents were of analytical grade.

### 2.2. Animals and Experimental Design

A total of 140 healthy adult male Sprague–Dawley (SD) rats (9–12 weeks old; body weight 240–260 g) were obtained from Jinan Pengyue Experimental Animal Breeding Co., Ltd. (Jinan, China). The animals were randomly allocated into 28 cages (5 rats per cage) and housed under specific pathogen-free conditions at the Animal Experiment Center of Anhui Agricultural University (Hefei, China). The facility maintained a controlled environment (temperature 22 ± 2 °C; relative humidity 50–60%; 12 h light/dark cycle). Following a 7-day acclimatization period, the rats were fasted for 12 h prior to the experiment, with free access to water.

Rats were randomly allocated to treatment groups using a random number table generated in Microsoft Excel 2007 (*n* = 5 per group) and administered via intragastric gavage either as NaF alone (control) or NaF combined with EGCG, TFs, TRs, TB, TPS, Ca, or Al. All test compounds were dissolved or suspended in 0.9% saline solution immediately before administration. The F concentration in all treatment groups was standardized to 40 mg/L, with each rat receiving a total volume of 5 mL. Blood samples (0.5 mL) were collected from the tail vein at 5, 10, 15, 30, 60, 120, 240, and 480 min post-administration [16]. Samples were allowed to clot at room temperature for 30 min and then centrifuged at 3000 rpm for 5 min at 4 °C for no more than 24 h before F analysis. The resulting plasma was separated and stored at 4 °C until analysis. Rats were euthanized at the end of the experiment (480 min), and fecal samples were collected directly from the colon. Feces were dried at 105 °C to a constant weight, then ground into a fine powder, and stored in sealed containers at room temperature until F analysis within 7 days. To minimize potential bias, the investigators performing the animal procedures (gavage, blood sampling, euthanasia) were different from those conducting the sample analysis (F measurement). The analysts were blinded to group allocation during all sample measurements and data analysis. The animal study was approved by the Ethics Committee of Anhui Agricultural University (protocol code: AHAU2020005, 20 March 2020).

The sample size per group was determined based on previous pharmacokinetic studies with similar designs [17]. The doses of EGCG (150–450 mg/kg) were selected based on previously published studies [18,19] and converted to human-equivalent doses using body surface area normalization. For example, the high dose of EGCG (450 mg/kg in rats) corresponds to approximately 70 mg/kg in humans, which is equivalent to the estimated daily intake from 8 to 10 cups of green tea based on an average EGCG content of 50–100 mg per cup [20]. Similarly, the doses of TFs, TRs, TB (200–800 mg/kg) and TPS (25–250 mg/kg) reflect ranges that could be achieved through regular consumption of fermented teas such as black tea, Pu-erh tea, and brick tea, based on their typical concentrations in tea leaves and infusion rates [21,22]. The doses of Ca and Al (800–3200 µg/kg) represent physiologically relevant levels comparable to those found in tea infusions and co-ingested with food [23,24].

### 2.3. F Determination

#### 2.3.1. Preparation of TISAB II

Total Ionic Strength Adjustment Buffer (TISAB II) was prepared by dissolving 68 g of Na_3_C_6_H_5_O_7_·2H_2_O and 58 g of NaCl in approximately 700 mL of deionized water. Then, 57 mL of CH_3_COOH was added, and the pH was adjusted to 5.0–5.2 using 10 mol/L NaOH. The solution was subsequently diluted to a final volume of 1000 mL with ultrapure water and stored at 4 °C until use.

#### 2.3.2. Determination of F Concentration in Rats Plasmas

A total of 100 μL of plasma was mixed with an equal volume of TISAB II buffer solution, and then the F concentration was measured and calculated using the F ion-selective electrode (9609 BNWP, Thermo, Waltham, MA, USA). The electrode is calibrated directly using the accompanying fluoride calibration solutions (0.1, 0.5, 1.0, 5.0, and 10.0 mg/L). Calibration curves were constructed by plotting millivolt readings against log-transformed F concentrations, and a new calibration was performed every 20 samples [25].

#### 2.3.3. Determination of F Concentration in Rat Feces

Total F content in feces was determined using the alkali fusion method [26]. Briefly, 0.10 g of dried fecal sample was placed in a 50 mL nickel crucible and moistened with a small amount of distilled water. Then, 3 mL of NaOH (16.7 mol/L) was added and mixed thoroughly. The crucible was placed in a muffle furnace and ashed using the following program: 150 °C for 60 min → 300 °C for 10 min → 600 °C for 30 min. After cooling, 20 mL of ultrapure water (90 °C) was added to dissolve the fused material. The pH of the solution was adjusted to 8–9 using 16.7 mol/L NaOH or 10% HCl, as needed. The volume was then brought to 50 mL with ultrapure water, mixed well, and filtered through medium-speed qualitative filter paper. Finally, equal volumes of the filtrate and TISAB II were mixed (1:1, *v*/*v*), and the F concentration was measured using the fluoride ion-selective electrode as described above.

### 2.4. Pharmacokinetic Analysis

Pharmacokinetic parameters were calculated using the PK Solver add-in program for Microsoft Excel [27]. The following parameters were determined:

T_max_: Time to reach maximum plasma concentration (min), an indicator of absorption rate.

C_max_: Maximum plasma concentration (mg/L), reflecting the extent of absorption and potential peak exposure.

T_1/2_: Elimination half-life (min), representing the time required for plasma concentration to decrease by half, which reflects the rate of elimination (metabolism plus excretion).

AUC_0–t_: Area under the plasma concentration–time curve from time 0 to the last measured time point (480 min) (mg·min/L), indicating the total F exposure and overall bioavailability.

### 2.5. Data Analysis

All data were expressed as the mean ± standard error of the mean (Mean ± SEM). Statistical analyses were performed using SPSS version 27.0 (IBM Corp., Armonk, NY, USA). Differences between groups were assessed using one-way analysis of variance (ANOVA), followed by the Tukey multiple comparison test or by Student’s *t*-test for comparisons between two groups. A *p* < 0.05 was considered statistically significant. Graphs were generated using Prism 9 (GraphPad Software, San Diego, CA, USA).

## 3. Results

The primary research question was whether each tea component, at physiologically relevant doses, would significantly alter F pharmacokinetic parameters (C_max_ and AUC_0-t_) compared to the control. Secondary exploratory analyses included examining dose-dependent effects within each component group and assessing correlations between plasma and fecal F data.

### 3.1. Effects of EGCG on F Metabolism in Rats

**Figure 1:**
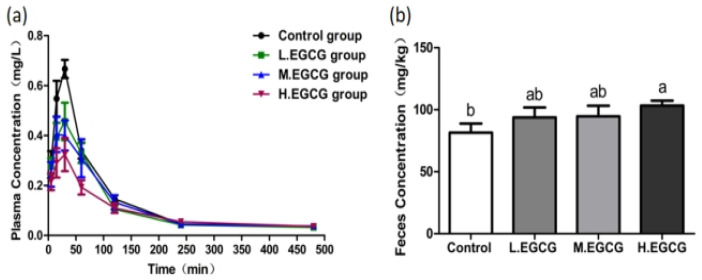
The effect of EGCG treatment at different concentrations on F content in rats. (**a**) The effect of EGCG treatment at different concentrations on plasma F metabolism in rats. (**b**) The effect of EGCG treatment at different concentrations on feces F in rats. EGCG concentrations were 150 mg/kg (LG), 250 mg/kg (MG), and 450 mg/kg (HG). One-way ANOVA combined with the Tukey multiple comparison test or Student’s t-test was employed to determine the significance of the data, presented as the Mean ± SEM (*n* = 5). Different letters represent significant differences between different treatment groups (*p* < 0.05).

As shown in Figure 1a, plasma F concentrations in all EGCG-treated groups were lower than those in the control group throughout the 480 min observation period, with levels gradually decreasing and stabilizing after 4 h. Fecal F excretion increased in a dose-dependent manner following EGCG administration (Figure 1b), and only the high-dose EGCG group (HG, 450 mg/kg) exhibited significantly higher fecal F content compared to the control group (*p* < 0.05).

Pharmacokinetic analysis (Table 1) revealed that EGCG treatment did not significantly alter T_max_ values relative to the control group (*p* > 0.05), indicating that EGCG does not affect the absorption rate of F. However, C_max_ values decreased progressively with increasing EGCG concentration. A significant reduction was observed only in the HG group (*p* < 0.05). These results indicated that high-dose EGCG effectively reduced the extent of F absorption and total systemic exposure in rats, which might have implications for reducing the risk of F toxicity, although direct toxicity endpoints (e.g., tissue pathology, behavioral changes) were not assessed in this study. Although T_1/2_ values were prolonged in all EGCG-treated groups, the differences were not statistically significant (*p* > 0.05), indicating a limited effect of EGCG on F elimination rate. Notably, the AUC_0–t_ value was significantly reduced in the HG group compared to the control (*p* < 0.05), confirming that high-dose EGCG treatment decreases total F exposure and bioavailability in rats.

### 3.2. Effects of Theapigments on F Metabolism in Rats

Theapigments, including TFs, TRs, and TB, are oxidation products of TPs [28]. Their effects on F metabolism were evaluated at three doses (low, LG; medium, MG; high, HG; 200, 400, and 800 mg/kg, respectively).

#### 3.2.1. Effects of TFs on F Metabolism in Rats

**Figure 2:**
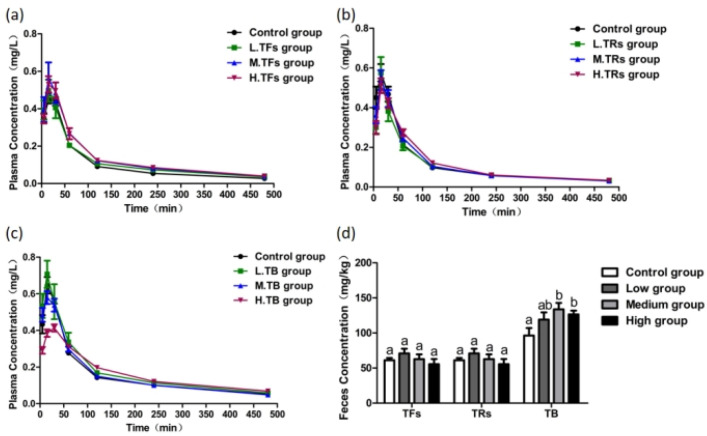
The effect of theapigment treatment at different concentrations on F content in rats: (**a**) the effect of TF treatment at different concentrations on plasma F metabolism in rats; (**b**) the effect of TR treatment at different concentrations on plasma F metabolism in rats; (**c**) the effect of TB treatment at different concentrations on plasma F metabolism in rats; (**d**) the effect of theapigment treatment at different concentrations on feces F in rats. theapigments concentrations were 200 mg/kg (LG), 400 mg/kg (MG), and 800 mg/kg (HG). One-way ANOVA combined with the Tukey multiple comparison test or Student’s *t*-test was employed to determine the significance of the data, presented as the Mean ± SEM (*n* = 5). Different letters represent significant differences between different treatment groups (*p* < 0.05).

Following TF administration, plasma F concentrations at 30 min followed the order HG > MG > LG > control, with levels gradually declining over time in all groups, (Figure 2a). No significant differences in fecal F content were observed between TF-treated groups and the control (Figure 2d, *p* > 0.05). Pharmacokinetic parameters (Table 2) showed no significant changes in T_max_, C_max_, or T_1/2_ across all TFs doses (*p* > 0.05). However, AUC_0–t_ values were significantly increased in the MG and HG groups (*p* < 0.05), indicating that medium and high doses of TFs enhance F bioavailability without affecting its absorption rate, extent, or elimination.

#### 3.2.2. Effects of TRs on F Metabolism in Rats

Plasma F concentrations in TR-treated groups converged with the control by 240 min (Figure 2b), and fecal F content remained unchanged (Figure 2d, *p* > 0.05). Pharmacokinetic analysis (Table 2) revealed no significant differences in any parameters across all TRs doses (*p* > 0.05), demonstrating that TRs have no measurable effect on F absorption, elimination, or bioavailability.

#### 3.2.3. Effects of TB on F Metabolism in Rats

In the TB-treated groups, plasma F dynamics exhibited a dose-dependent pattern. The HG group showed lower plasma F levels than the control during the first 60 min post-administration, followed by higher levels between 60 and 240 min (Figure 2c), suggesting a delayed absorption effect. Fecal F excretion was significantly increased in the MG and HG groups (Figure 2d, *p* < 0.05), indicating that TB promotes fecal F elimination. Pharmacokinetic analysis (Table 2) confirmed these observations: the HG group exhibited significantly prolonged T_max_ and reduced C_max_ (*p* < 0.05), while AUC_0–t_ values remained unchanged (*p* > 0.05). These results demonstrate that high-dose TB effectively slows F absorption and reduces its peak plasma concentration, thereby decreasing F bioavailability.

### 3.3. Effects of TPS on F Metabolism in Rats

**Figure 3:**
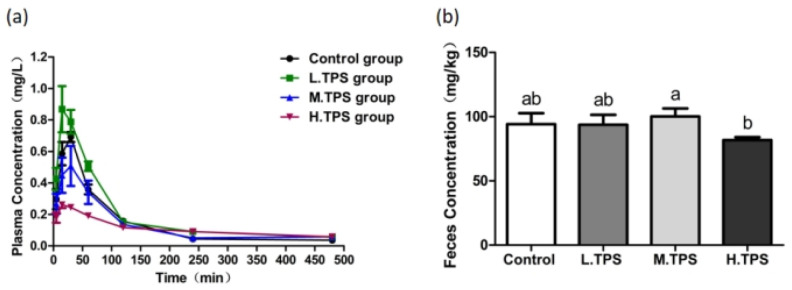
The effect of TPS treatment at different concentrations on F content in rats. (**a**) The effect of TPS treatment at different concentrations on plasma F metabolism in rats. (**b**) The effect of TPS treatment at different concentrations on feces F in rats. TPS concentrations were 25 mg/kg (LG), 100 mg/kg (MG), and 250 mg/kg (HG). One-way ANOVA combined with the Tukey multiple comparison test or Student’s *t*-test was employed to determine the significance of the data, presented as the Mean ± SEM (*n* = 5). Different letters represent significant differences between different treatment groups (*p* < 0.05).

Plasma F profiles following TPS administration are presented in Figure 3a. During the first 120 min, the LG group (25 mg/kg) showed higher plasma F concentrations than the control, whereas the MG (100 mg/kg) and HG (250 mg/kg) groups exhibited lower levels. Fecal F excretion was elevated in the MG group but reduced in the HG group compared to the control (Figure 3b). However, these differences were not statistically significant (*p* > 0.05). Compared with the MG, the fecal F concentration in the HG group was significantly lower (*p* < 0.05). It is speculated that there is a dose-dependent regulatory effect of TPS on F elimination.

Pharmacokinetic analysis (Table 3) revealed no significant differences in T_max_ or T_1/2_ across all TPS doses (*p* > 0.05), indicating that TPS does not alter F absorption or elimination rates. However, C_max_ and AUC_0–t_ were significantly reduced in the HG group (*p* < 0.05), demonstrating that high-dose TPS effectively decreases both peak F absorption and total systemic F exposure, thereby reducing F bioavailability.

### 3.4. Effects of Mineral Elements on F Metabolism in Rats

#### 3.4.1. Effects of Ca on F Metabolism in Rats

**Figure 4:**
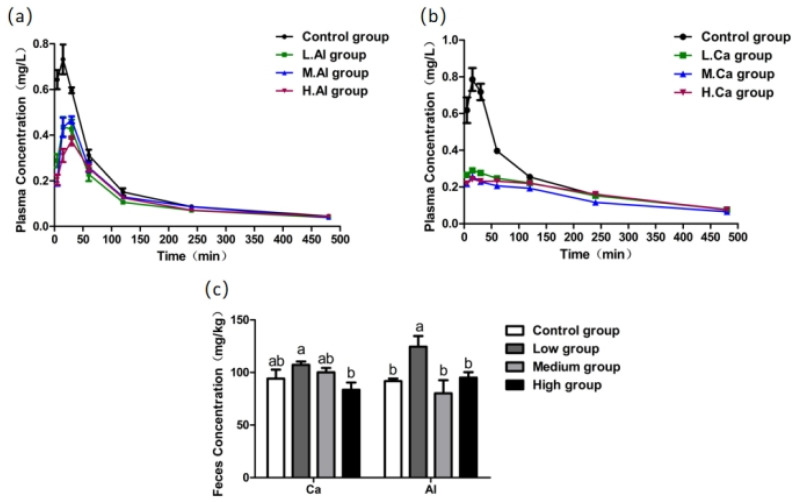
The effect of mineral element treatment at different concentrations on F content in rats: (**a**) the effect of Al treatment at different concentrations on plasma F metabolism in rats; (**b**) the effect of Ca treatment at different concentrations on plasma F metabolism in rats; (**c**) the effect of Al and Ca treatment at different concentrations on feces F in rats. Mineral elements concentrations were 800 µg/kg (LG), 1600 µg/kg (MG), and 3200 µg/kg (HG). One-way ANOVA combined with the Tukey multiple comparison test or Student’s *t*-test was employed to determine the significance of the data, presented as the Mean ± SEM (*n* = 5). Different letters represent significant differences between different treatment groups (*p* < 0.05).

Following Ca administration, plasma F concentrations in all treatment groups were consistently lower than those in the control group during the 0–120 min period (Figure 4b). The MG group (1600 µg/kg) maintained the lowest plasma F levels after 60 min. Fecal F excretion was increased in the LG (800 µg/kg) and MG groups (Figure 4c), with only the LG group showing the significantly effect (*p* < 0.05). Pharmacokinetic analysis (Table 4) showed no significant changes in T_max_ or T_1/2_ (*p* > 0.05), but C_max_ and AUC_0–t_ were significantly reduced across all Ca doses (*p* < 0.05). These findings indicate that Ca treatment effectively reduces F absorption and total exposure without affecting its absorption or elimination rates, thereby decreasing F bioavailability.

#### 3.4.2. Effects of Al on F Metabolism in Rats

Plasma F concentrations in all Al-treated groups were lower than those in the control throughout the 0–240 min period (Figure 4a). The LG group (800 µg/kg) exhibited a reduction, particularly between 60 and 240 min. Fecal F excretion was significantly increased only in the LG group (Figure 4c, *p* < 0.05). Pharmacokinetic analysis (Table 4) revealed no significant changes in T_max_ or T_1/2_ (*p* > 0.05), while C_max_ was significantly reduced across all Al doses (*p* < 0.05). AUC_0–t_ values were also decreased, with significant reductions in the LG and HG groups (*p* < 0.05). These results demonstrate that Al treatment reduces F absorption and total exposure, particularly at low and high doses, without affecting absorption or elimination kinetics.

### 3.5. Comparative Overview of Component Effects on F Pharmacokinetics

To facilitate cross-component comparison of the effects on F bioavailability, the key findings for all eight tea components are summarized in Table 5. This overview integrates the dose-dependent responses of each component across the primary pharmacokinetic parameters (C_max_, AUC_0–t_, T_max_, T_1/2_) and fecal F excretion. TB, Ca, and Al consistently reduced C_max_ and AUC_0–t_ while promoting fecal F excretion, indicating their potential to decrease F bioavailability. For TB, this effect was accompanied by prolonged T_max_, suggesting delayed absorption. For Ca and Al, T_max_ and T_1/2_ remained unchanged, consistent with a precipitation-based mechanism in the intestinal lumen. TFs did not alter absorption kinetics but significantly increased AUC_0–t_ at medium and high doses, suggesting enhanced total F exposure without changes in peak concentration or elimination rate. This unexpected finding warrants further investigation. EGCG and TPS showed clear dose–response relationships. High doses of both components reduced C_max_ and AUC_0–t_, while low doses had minimal or opposite effects. These findings highlight the importance of dose consideration in interpreting the net impact of these components. TRs showed no significant effects on any pharmacokinetic parameters or fecal F excretion across all doses, indicating minimal interaction with F under the current experimental conditions. This integrated summary underscores that the modulation of F bioavailability by tea components is component-specific and dose-dependent, with some constituents offering protective potential (TB, Ca, Al) and others unexpectedly increasing F exposure (TFs). These patterns provide a foundation for understanding how different tea types, varying in their component profiles, may differentially influence F bioavailability.

## 4. Discussion

This study systematically evaluated the effects of major tea components—EGCG, theapigments (TFs, TRs, TB), TPSs, and mineral elements (Ca, Al)—on F metabolism in rats. Our findings revealed that different tea constituents exert distinct, dose-dependent effects on F bioavailability through diverse mechanisms. While the present study provided the first comparative pharmacokinetic profiling of these components under identical conditions, several of the mechanistic interpretations discussed below are speculative and require experimental validation in future studies.

### 4.1. EGCG Reduced F Exposure by Inhibiting Intestinal Absorption

EGCG, the predominant catechin in tea, has been shown to modulate intestinal metabolism and the bioavailability of various chemical substances [29]. Our results demonstrated that high-dose EGCG (450 mg/kg) significantly reduced C_max_ by 61.76% and AUC_0–t_ by 37.48% while increasing fecal F excretion by 26.79%, with no change in T_1/2_. This pattern suggests that EGCG primarily inhibits intestinal F absorption rather than accelerating systemic elimination. A plausible mechanism involves enhancement of intestinal barrier function and reduction in paracellular passive transport, as previously reported for other compounds [30]. However, this interpretation remains hypothetical, as we did not directly assess barrier function or transport pathways. Consistent with our findings, previous studies have reported that polyphenols could bind F through hydrogen bonding [12], which might contribute to reduced absorption. Notably, the EGCG doses used in this study (150–450 mg/kg) correspond to human-equivalent intakes of approximately 24–70 mg/kg [18], achievable through regular consumption of green tea (based on an average EGCG content of 50–100 mg per cup) [31]. Nevertheless, in fermented teas, EGCG content is substantially diminished due to oxidative polymerization during processing [32], suggesting that its direct contribution to F regulation in dark teas is limited. In such teas, oxidative polymerization products—particularly theapigments—likely play a more critical role.

### 4.2. Theapigments: Structural Diversity Determines Functional Divergence in F Regulation

During full fermentation and post-fermentation, tea polyphenols undergo extensive oxidation to form theapigments [21]. Our study revealed marked structure-dependent effects of theapigments on F bioavailability.

TFs did not alter F absorption kinetics (T_max_ and C_max_ unchanged) but significantly increased total F exposure, as evidenced by an elevated AUC_0–t_. This unexpected finding suggested that TFs might not affect initial F uptake but could interfere with its tissue distribution or renal excretion, leading to prolonged retention in systemic circulation. However, this interpretation is speculative, as we did not measure tissue F concentrations or urinary excretion. And this may be the first report of TFs potentially increasing F bioavailability, a finding that warrants confirmation in independent studies and further investigation into the underlying mechanisms. TRs showed no significant effects on any pharmacokinetic parameters or fecal F excretion, indicating minimal interaction with F at the pharmacokinetic level within this experimental system. This result is consistent with the high molecular weight and complex polymeric structure of TRs, which may limit their ability to interact with small ions like F.

In contrast, TB exhibited the most pronounced protective effects against F bioavailability. High-dose TB (800 mg/kg) significantly delayed F absorption (T_max_: 18 → 30 min), reduced C_max_ by 35.38%, and promoted fecal F excretion. This multifunctional effect may be attributed to several potential mechanisms. First, TB may form insoluble or macromolecular complexes with F in the intestinal lumen, reducing its solubility and physically hindering absorption—a mechanism analogous to that proposed for other high-molecular-weight polyphenols [33,34]. Second, TB’s known bioactivities, including modulation of gut microbiota and interference with enterohepatic circulation [35,36], could contribute to trapping F and facilitating its fecal elimination. However, these mechanisms remain hypothetical and require direct experimental validation through in vitro binding assays and microbiota analysis. These findings provide a potential mechanistic basis for the lower F bioavailability risk associated with TB-rich dark teas, such as ripe Pu-erh tea and brick tea.

### 4.3. TPS Exhibited Dose-Dependent Bidirectional Effects on F Bioavailability

TPSs have demonstrated anti-inflammatory effects in models of inflammatory bowel disease [22], and recent evidence suggests that TPS can alleviate F-induced colitis in rats [37]. Our study revealed a clear dose–response relationship in TPS-mediated modulation of F metabolism. At low doses (25 mg/kg), TPS slightly increased plasma F concentrations, potentially through formation of soluble complexes that facilitate transport. At high doses (250 mg/kg), however, TPS significantly reduced C_max_ by 51.72% and AUC_0–t_ by 24.38%. This bidirectional effect suggests a dynamic equilibrium in TPS-F interactions, possibly involving a shift from soluble complex formation at low concentrations to stable insoluble aggregation at high concentrations. However, this hypothesis requires confirmation through physicochemical characterization of TPS-F complexes across a range of concentrations. The dose-dependent nature of this effect has important implications for interpreting the net impact of TPS-containing teas on F bioavailability, as the outcome may vary with the polysaccharide content of different tea types and brewing conditions.

### 4.4. Mineral Elements: Efficient Precipitation as a Key Immobilization Mechanism

The formation of fluoro-aluminum complexes and calcium F precipitates has been well documented in water defluoridation studies [38,39]. Our results extend these findings to the in vivo setting, demonstrating that both Ca and Al significantly reduced C_max_ by 39.19–69.62% and AUC_0–t_ while increasing fecal F excretion, without affecting T_max_ or T_1/2_. This pattern strongly implicates a classic chemical precipitation mechanism involving the formation of insoluble complexes, such as CaF_2_ or AlFₓ species, within the intestinal lumen [40,41]. These complexes exhibit extremely low solubility under physiological pH conditions, effectively sequestering F ions and promoting their physical elimination via feces. These findings confirm that mineral elements naturally present in tea, as well as those co-ingested with diet, are critical determinants of F bioavailability. Notably, the doses used in this study (800–3200 µg/kg) are physiologically relevant [23,24], corresponding to levels achievable through consumption of mineral-rich teas and typical diets.

### 4.5. Integrated Perspective and Implications for Human Tea Consumption

Collectively, our findings demonstrated that the regulation of F bioavailability by tea components involved multiple constituents acting through distinct mechanisms. EGCG inhibited intestinal absorption; theapigments exhibited structure-dependent effects ranging from neutral (TRs) to potentially adverse (TFs) to strongly protective (TB); TPS provided dose-dependent modulation; and mineral elements contributed fundamental chemical precipitation mechanisms. Together, these components collectively determine the net bioavailability of F following tea consumption. These findings have several implications for consumers, particularly in regions where high-F tea consumption is common. First, our results suggested that the type of tea consumed may influence F bioavailability. Teas rich in TB (e.g., ripe Pu-erh tea, brick tea) or containing high levels of Ca and Al may reduce F absorption, potentially mitigating fluorosis risk. Conversely, teas high in TFs (e.g., black tea) could theoretically increase F exposure, although this finding requires confirmation in human studies and should be interpreted cautiously. Second, the dose-dependent effects of TPS suggested that moderate consumption of certain teas might offer protective effects, while excessive intake could have different outcomes. Based on our rat data, a human consuming 4–5 cups of TB-rich tea daily might experience a 30–40% reduction in F bioavailability compared to consuming the same amount of TF-rich tea. However, these estimates are preliminary and require direct validation in human clinical trials.

### 4.6. Limitations and Future Directions

Several limitations of this study should be acknowledged. First, the sample size (*n* = 5 per group) was relatively small, which may limit statistical power to detect small effect sizes. Second, only male rats were used to avoid hormonal confounding, but this limits generalizability to female populations; future studies should include both sexes to assess potential sex differences in F metabolism. Third, we measured only fecal F excretion and did not collect urine, which is the primary route of F elimination in mammals. The absence of urinary data prevents complete mass balance calculations and may underestimate total F clearance. Fourth, this was an acute exposure study using single doses, whereas real-world tea consumption involves chronic, repeated exposure. Chronic studies are needed to assess whether the observed effects persist, attenuate, or amplify over time. Fifth, the pharmacokinetic profile was truncated at 480 min; although this covers the absorption and distribution phases, longer monitoring (24–48 h) would better characterize elimination kinetics. Sixth, the mechanistic interpretations proposed throughout this discussion (e.g., complex formation, microbiota modulation, interference with renal excretion) are speculative and require direct experimental validation through in vitro binding assays, urinary F measurements, tissue distribution analysis, and gut microbiome profiling. Finally, while we selected doses to be physiologically relevant based on human-equivalent calculations, direct extrapolation to human tea consumption requires confirmation in controlled human studies.

Future research should address these gaps as follows: (1) including urinary F measurements to enable complete mass balance calculations; (2) conducting chronic exposure studies to assess long-term effects; (3) performing in vitro binding assays to characterize F interactions with individual tea components; (4) investigating potential sex differences in F metabolism; and (5) validating key findings in human clinical trials. Such studies will strengthen the translational relevance of our findings and inform evidence-based dietary recommendations for fluoride risk mitigation.

## 5. Conclusions

This study provided the first systematic, comparative pharmacokinetic profiling of eight major tea components, including EGCG, TFs, TRs, TB, TPS, Ca, and Al, on F metabolism in rats. Our findings demonstrate that tea components exerted distinct, dose-dependent effects on F bioavailability. Specifically, TB, Ca, and Al consistently reduced F absorption and promoted fecal excretion, suggesting protective potential against F overexposure. In contrast, TFs unexpectedly increased total F exposure, a finding that warrants further investigation. EGCG and TPS showed dose-dependent effects, with high doses reducing F bioavailability, while TRs had minimal impact. These results advance understanding of how different tea types, varying in their component profiles, may differentially influence F bioavailability. The findings have implications for assessing the safety of high-F teas and for developing dietary strategies to mitigate fluorosis risk. However, the study’s scope is limited to acute exposure in male rats, and direct extrapolation to human tea consumption requires confirmation in clinical studies. Future research should focus on validating the proposed mechanisms through in vitro binding assays, incorporating urinary F measurements, conducting chronic exposure studies, and investigating potential sex differences in F metabolism.

## Figures and Tables

**Table 1:** Pharmacokinetic parameters of EGCG treatments at different concentrations on F metabolism in rats.

Parameters	Control	LG	MG	HG
T_max_ (min)	30.00 ± 0.00 a	33.75 ± 13.12 a	33.75 ± 13.12 a	30.00 ± 15.00 a
C_max_ (mg/L)	0.68 ± 0.05 a	0.51 ± 0.06 ab	0.48 ± 0.09 ab	0.26 ± 0.06 b
T_1/2_ (min)	108.10 ± 1.02 a	147.00 ± 70.74 a	129.84 ± 25.69 a	309.31 ± 115.18 a
AUC_0–t_ (mg·min/L)	64.14 ± 4.72 a	49.41 ± 3.98 ab	60.68 ± 11.11 ab	40.10 ± 2.84 b

Note: EGCG concentrations were 150 mg/kg (LG), 250 mg/kg (MG), and 450 mg/kg (HG). One-way ANOVA combined with the Tukey multiple comparison test or Student’s *t*-test was employed to determine the significance of the data, presented as the Mean ± SEM (*n* = 5). Different letters represent significant differences between different treatment groups (*p* < 0.05).

**Table 2:** Pharmacokinetic parameters of theapigment treatments at different concentrations on F metabolism in rats.

Treatments	Groups	T_max_ (min)	C_max_ (mg/L)	T_1/2_ (min)	AUC_0_**_–_**_t_ (mg·min/L)
TFs	Control	18.00 ± 6.70 a	0.45 ± 0.04 a	214.47 ± 42.37 a	48.38 ± 2.54 a
	LG	15.00 ± 0.00 a	0.49 ± 0.13 a	228.56 ± 21.48 a	54.42 ± 8.13 ab
	MG	18.00 ± 6.70 a	0.56 ± 0.17 a	219.46 ± 8.66 a	63.27 ± 8.64 b
	HG	15.00 ± 0.00 a	0.53 ± 0.09 a	218.32 ± 14.62 a	63.61 ± 8.06 b
TRs	Control	15.00 ± 0.00 a	0.57 ± 0.09 a	222.79 ± 35.90 a	53.33 ± 3.95 a
	LG	18.00 ± 6.70 a	0.57 ± 0.17 a	210.67 ± 26.06 a	54.42 ± 8.13 a
	MG	18.00 ± 6.70 a	0.53 ± 0.11 a	211.71 ± 7.33 a	50.87 ± 6.18 a
	HG	15.00 ± 0.00 a	0.51 ± 0.08 a	184.50 ± 39.38 a	56.13 ± 4.47 a
TB	Control	18.00 ± 6.70 a	0.65 ± 0.10 a	247.69± 23.60 a	73.52 ± 2.40 a
	LG	15.00 ± 0.00 a	0.71 ± 0.16 ab	242.53 ± 36.37 a	83.18 ± 16.70 a
	MG	21.00 ± 8.22 a	0.60 ± 0.06 ab	220.51 ± 13.33 a	73.67 ± 4.46 a
	HG	30.00 ± 0.00 b	0.42 ± 0.04 b	242.87 ± 14.19 a	78.19 ± 4.98 a

Note: theapigments concentrations were 200 mg/kg (LG), 400 mg/kg (MG), and 800 mg/kg (HG). One-way ANOVA combined with the Tukey multiple comparison test or Student’s *t*-test was employed to determine the significance of the data, presented as the Mean ± SEM (*n* = 5). Different letters represent significant differences between different treatment groups (*p* < 0.05).

**Table 3:** Pharmacokinetic parameters of TPS treatments at different concentrations on F metabolism in rats.

Parameters	Control	LG	MG	HG
T_max_ (min)	24.00 ± 8.22 a	13.00 ± 4.47 a	15.00 ± 0.00 a	30.00 ± 21.21 a
C_max_ (mg/L)	0.58 ± 0.09 ab	0.72 ± 0.18 a	0.62 ± 0.15 ab	0.28 ± 0.04 b
T_1/2_ (min)	257.02 ± 88.52 a	255.15 ± 35.83 a	203.83 ± 58.54 a	244.72 ± 34.42 a
AUC_0–t_ (mg·min/L)	90.15 ± 12.48 a	85.08 ± 8.95 ab	85.48 ± 10.47 ab	68.17 ± 4.42 b

Note: TPS concentrations were 25 mg/kg (LG), 100 mg/kg (MG), and 250 mg/kg (HG). One-way ANOVA combined with the Tukey multiple comparison test or Student’s *t*-test was employed to determine the significance of the data, presented as the Mean ± SEM (*n* = 5). Different letters represent significant differences between different treatment groups (*p* < 0.05).

**Table 4:** Pharmacokinetic parameters of mineral element treatments at different concentrations on F metabolism in rats.

Treatments	Groups	T_max_ (min)	C_max_ (mg/L)	T_1/2_ (min)	AUC_0_**_–_**_t_ (mg·min/L)
Ca	Control	18.00 ± 6.70 a	0.79 ± 0.10 a	247.69 ± 11.65 a	108.83 ± 6.65 a
	LG	15.00 ± 0.00 a	0.29 ± 0.02 b	236.87 ± 17.03 a	79.58 ± 8.58 b
	MG	18.00 ± 6.70 a	0.25 ± 0.03 b	236.76 ± 13.19 a	65.29 ± 1.89 b
	HG	33.00 ± 22.50 a	0.24 ± 0.02 b	233.14 ± 17.12 a	78.01 ± 2.87 b
Al	Control	18.00 ± 6.70 a	0.74 ± 0.12 a	207.21 ± 20.92 a	75.85 ± 10.78 a
	LG	21.00 ± 8.21 a	0.45 ± 0.09 b	252.63 ± 64.92 a	54.29 ± 6.62 b
	MG	27.00 ± 6.70 a	0.50 ± 0.05 b	202.97 ± 47.95 a	69.48 ± 7.50 ab
	HG	24.00 ± 8.21 a	0.37 ± 0.04 b	212.40 ± 56.18 a	54.51 ± 3.48 b

Note: Mineral elements concentrations were 800 µg/kg (LG), 1600 µg/kg (MG), and 3200 µg/kg (HG). One-way ANOVA combined with the Tukey multiple comparison test or Student’s *t*-test was employed to determine the significance of the data, presented as the Mean ± SEM (*n* = 5). Different letters represent significant differences between different treatment groups (*p* < 0.05).

**Table 5:** Summary of the effects of tea components on F pharmacokinetic parameters and fecal excretion.

Treatments	Groups	Cmax (mg/L)	AUC0–t (mg·min/L)	Tmax (min)	T1/2 (min)	Fecal F
EGCG	LG	—	—	—	—	—
	MG	—	—	—	—	—
	HG	↓	↓	—	—	↑
TFs	LG	—	—	—	—	—
	MG	—	↑	—	—	—
	HG	—	↑	—	—	—
TRs	LG	—	—	—	—	—
	MG	—	—	—	—	—
	HG	—	—	—	—	—
TB	LG	—	—	—	—	—
	MG	—	—	—	—	↑
	HG	↓	—	↑	—	↑
TPS	LG	—	—	—	—	—
	MG	—	—	—	—	—
	HG	↓	↓	—	—	—
Ca	LG	↓	↓	—	—	—
	MG	↓	↓	—	—	—
	HG	↓	↓	—	—	—
Al	LG	↓	↓	—	—	↑
	MG	↓	—	—	—	—
	HG	↓	↓	—	—	—

Note: “↑” indicates a significant increase compared to the control (*p* < 0.05), “↓” indicates a significant decrease compared to the control (*p* < 0.05), and “—“ indicates no significant change compared to the control. All comparisons are versus the respective control groups. LG, MG, HG correspond to low, medium, and high doses as defined in Section 3.1, Section 3.2, Section 3.3 and Section 3.4.

## Data Availability

The original contributions presented in the study are included in the article; further inquiries can be directed to the corresponding authors.

## Abbreviations

The following abbreviations are used in this manuscript:

F	Fluoride
EGCG	Epigallocatechin gallate
TRs	Thearubigins
TFs	Theaflavins
TB	Theabrownine
TPSs	Tea polysaccharides
TISAB	Total Ionic Strength Adjustment Buffer II
